# Supplementary material for: Vimentin-Positive Circulating Tumor Cells as Diagnostic and Prognostic Biomarkers in Patients with Biliary Tract Cancer
Source: J Clin Med. 2021 Sep 27;10(19):4435. doi: 10.3390/jcm10194435 (PMC8509386; doi:10.3390/jcm10194435)
Supplement: Supplementary file 1 [file jcm-10-04435-s001.zip › jcm-1332714-supplementary.pdf]

Supplementary Table S1. prognostic value according to V-CTC level in subgroup analysis

	V-CTC count < 50	V-CTC count > 50	p-value
GB cancer (n=8)	n=7	n=1	
Overall Survival (days)	324 (6.8-443.1)	138	0.353
IHCC (n=12)	n=9	n=3	
Overall Survival (days)	170 (0-345.3)	95 (0-224.6)	0.076
Klatskin (n=11)	n=9	n=2	
Overall Survival (days)	293 (141.1-444.9)	245	0.835
CBD cancer (n=21)	n=16	n=5	
Overall Survival (days)	307 (267.8-346.2)	218 (117.1-318.9)	0.072

V-CTC, Vimentin + CTC , GB (gallbladder), OS (overall survival), IHCC (intrahepatic cholangiocarcinoma), CBD (common bile duct)

Supplementary table S2. comparison of PFS according to CTC marker and CA19-9 in patients with surgery or chemotherapy who could checked the PFS

	low	High	p-value
	N=20	N=15	
CTC40	191 (151.6-230.4)	151 (109.3-192.7)	0.377
	N=18	N=17	
V-CTC15	191 (153.6-228.4)	167 (125.3-208.7)	0.349
	N=30	N=5	
V-CTC50	190 (140.3-239.6)	140 (107.8-172.2)	0.166
	N=18	N=17	
VCR40	190 (177.5-202.5)	167 (125.3-208.7)	0.664
	< 40 (n=12)	> 40 (n=23)	
CA19-9	284 (168.5-399.5)	163 (152.5-217.4)	0.011

Supplementary Table S3. Baseline characteristics between BTC patients with CTC detected and whose without CTC detection.

	CTC not detection (n=7)	CTC detection (n=52)	p-value
Sex male, (%)	5 (71.4)	32 (61.5)	0.619
Age	74.0 ± 6.9	69.2 ± 10.8	0.257
Diagnosis GB/IHCC/EHCC/Klatskin	1(14.3)/2(28.6)/3(42.9)/1(14.3)	8(15.4)/12(23.1)/21(40.4)/11(21.2)	0.798
hepatitis HBV/HCV	1 (14.3) / 0 (0)	3 (5.8) / 1 (1.9)	0.748
LC	0 (0)	1 (1.9)	0.717
Hypertension	2 (28.6)	17 (32.7)	0.830
Diabetes	0 (0)	10 (19.2)	0.210
smoking none/current/ex-	4 (57.1)/ 1 (14.3)/ 2 (28.6)	40 (76.9)/ 8 (15.4)/ 4 (7.7)	0.129
alcoholic	0 (0)	14 (26.9)	0.120
Dyslipidemia	1 (14.3)	5 (9.6)	0.707
BMI	23.9 ± 3.9	22.8 ± 3.0	0.378
Pathology well-/moder-/poor	0 (0) / 3 (42.9) / 0 (0)	4 (7.7) / 19 (36.5) / 9 (17.3)	0.508
Metastatic	5 (71.4)	12 (23.1)	0.007*
Operable	2 (28.6)	33 (63.5)	0.080
**Laboratory findings**			
WBC	9835.7 ± 4486.5	7492.1 ± 3739.5	0.134
NLR	18.7 ± 25.2	4.1 ± 4.3	<0.001*
Hb	12.8 ± 1.9	12.4 ± 1.7	0.612
PLT (k)	187.0 ± 76.9	270.3 ± 85.3	0.017*
ALT	188.3 ± 186.8	119.9 ± 125.0	0.206
ALP	421.5 ± 194.1	326.7 ± 263.1	0.362
Total Bilirubin	8.59 ± 11.10	5.76 ± 7.70	0.391
Albumin	3.76 ± 1.06	4.00 ± 0.54	0.342
PNI	43.0 ± 13.9	47.6 ± 6.5	0.139
BUN	18.8 ± 9.5	14.4 ± 5.2	0.064
Creatinine	0.87 ± 0.30	0.79 ± 0.21	0.256
C-related protein	5.5 ± 6.6	2.5 ± 3.9	0.090
CEA	9.2 ± 11.4	6.5 ± 9.8	0.564
CA19-9	4475.7 ± 6694.9	701.3 ± 1240.2	<0.001*

BTC (biliary tract cancer), CTC (circulating tumor cell), HBV (hepatitis B virus), HCV (hepatitis C virus), LC (liver cirrhosis), NLR (neutrophil/lymphocyte ratio), PNI (prognostic nutrition index),

Supplementary Figure S1. ROC curve of CTC, V-CTC, VCR

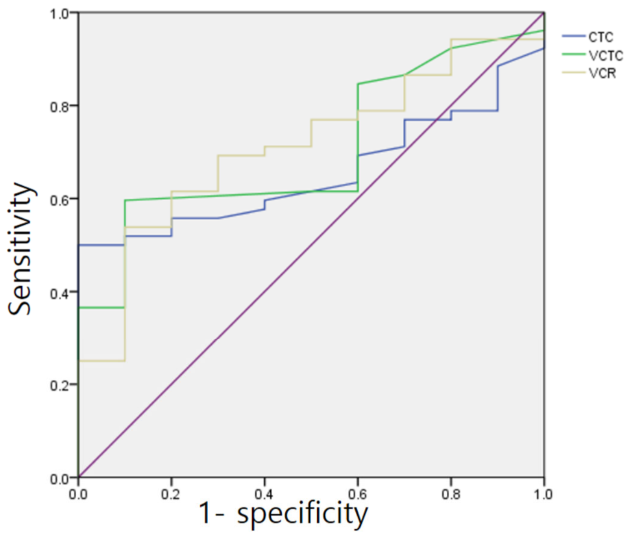

	AUC	Sensitivity	Specificity
CTCs	0.654	55.85	80%
V-CTCS	0.704	61.5%	50%
VCR	0.712	48.1%	90%
